# The Motivational Hierarchy between the Personal Self and Close Others in the Chinese Brain: an ERP Study

**DOI:** 10.3389/fpsyg.2016.01467

**Published:** 2016-09-27

**Authors:** Xiangru Zhu, Lili Wang, Suyong Yang, Ruolei Gu, Haiyan Wu, Yuejia Luo

**Affiliations:** ^1^Institute of Psychology and Behavior, Henan University, KaifengChina; ^2^School of Educational Science, Huaiyin Normal University, HuaianChina; ^3^Department of Psychology, Shanghai University of Sport, ShanghaiChina; ^4^Institute of Psychology, Chinese Academy of Sciences, BeijingChina; ^5^Institute of Affective and Social Neuroscience, Shenzhen University, ShenzhenChina

**Keywords:** personal self, close others, motivational hierarchy, feedback-related negativity (FRN), event-related potential (ERP)

## Abstract

People base their decisions not only on their own self-interest but also on the interests of close others. Generally, the personal self has primacy in the motivational hierarchy in the Western culture. A recent study found that friends have the same motivational hierarchy as the personal self in the Eastern collectivist culture. Remaining unknown is whether the motivational hierarchy of the personal self and close others can be manifested in the collectivist brain. In the present study, we asked participants to gamble for the personal self, close others (i.e., mother, father, and close friend), and strangers. The positive-going deflection of event-related potentials (ERPs) in response to positive feedback showed the following pattern: personal self = mother = father > friend > stranger. In the loss condition, no significant beneficiary effect was observed. The present results indicate that the personal self and parents are intertwined in the motivational system in the Chinese undergraduate student brain, supporting the view that the personal self and parents have the same motivational primacy at the electrocortical level.

## Introduction

Human behavior is guided by motivation. Self-interest is the cardinal human motivation. Humans should be and are motivated by self-interest (Miller, 1999). Self-interest appears to explain most of what people want and do. However, humans are social animals. In some cases, we make money not only for ourselves but also for our parents and friends. When making money for ourselves, parents, or friends, one question arises: who is most important? The hierarchy of the self-motivation system can be studied at both the behavioral and electrocortical levels (Kitayama and Park, 2014). To provide a comprehensive understanding of the motivational hierarchy among the personal self, parents, and friends, the present study evaluated event-related potentials (ERPs) during a gambling task to investigate the hierarchy of the self-motivation system in Chinese college students.

A widely accepted notion is that Asians are collectivist, with the self identified within an in-group, whereas Westerners are individualists, with the self distinct from the in-group. East Asians emphasize the interconnectedness of human beings and contingencies between individual behavior and the thoughts and actions of others in social relationships (Markus and Kitayama, 1991). Previous studies have used a trait judgment paradigm and interpreted medial prefrontal cortex (mPFC) activation as an indicator of the neural representation of the self and close others (Kelley et al., 2002; Seger et al., 2004; Ochsner et al., 2005; Northoff et al., 2006; Zhu et al., 2007; Wu et al., 2010). With regard to family members, neuroimaging studies found that the results depend on cultural factors. Neuroimaging studies compared East Asians and Westerners and found that subjects from collectivistic cultures included representations of the mother within representations of the self (Zhu et al., 2007; Wang et al., 2012; Wuyun et al., 2014), whereas subjects from individualistic cultures did not (Heatherton et al., 2006; Vanderwal et al., 2008). For example, studies from China found that participants exhibited similar activation of regions of the mPFC when making judgments about themselves and their mothers.

Relationships with close others are necessary and associated with psychological and physical health benefits. However, remaining unclear is whether the personal self and close others are equally important or meaningful. Our previous study used a gambling paradigm and ERP technique to compare the motivational hierarchy between the personal self and mother. The feedback-related negativity (FRN) results showed that the self and mother had the same motivational hierarchy in the Chinese brain (Zhu et al., 2015b). This result is consistent with the same level of activation between the self and mother (Zhu et al., 2007).

Friends, at least to some extent, influence an individual’s development. However, the status of a friend in the self-motivational system is modulated by culture factors. Kitayama and Park (2014) performed a study with European Americans and Asians to investigate whether cultural differences in the self-motivational system is modulated by self-construals. They examined whether error-related negativity (ERN), a neural marker of the level of motivation can differentiate between the personal self and friends. The amplitude of ERN was larger in the personal self condition than in the friend condition in a Western culture but not in an Eastern collectivist culture. This result suggests that friends gain the same status as the personal self in the self-motivation system in Chinese culture.

However, previous studies left some unresolved questions about the self-motivation hierarchy. First, Kitayama and Park (2014) found that friends have the same motivational status as the personal self, for Chinese, but other behavioral studies found that friends were less important than their parents (Li, 2002; Cai et al., 2013). Therefore, still unclear is whether friends possess the same motivational hierarchy as the mother. Second, a recent study found that the father and mother are unequally represented in the mPFC in the brains of people from a collectivist culture and follow a general pattern of activation of self = mother > father (Wang et al., 2012). The difference in neural representations between the mother and father may modulate the motivational hierarchy, but no previous study has directly explored the motivational hierarchy of the personal self and parents.

The present study examined the motivational hierarchy of the personal self and close others. We compared FRN associated with outcome evaluation using a simple gambling task. In each trial, the beneficiary could be the personal self, the mother, the father, a friend, or a stranger. FRN is a medial frontal negative-trending component that peaks approximately 250 ms following feedback presentation and is a key component associated with outcome evaluation (Gehring and Willoughby, 2002). FRN has two separate but temporally comparable components: reward positivity, in response to rewards; and reward negativity in response to losses.

Influential theories have proposed that FRN reflects a reinforcement learning signal that is associated with prediction errors, especially when outcomes are worse than expected (Holroyd and Coles, 2002; Hajcak et al., 2007). This theory suggests that FRN is an index of the activity of the midbrain dopamine system, which evaluates the ongoing event as a binary “good–no good” dimension (Holroyd et al., 2006). Because FRN is tightly related to monetary loss and error feedback, it has typically been viewed as a negative deflection in the ERP waveform that increases in response to monetary loss and either decreases or is absent in response to monetary gain. Recent work has suggested the viewpoint that the amplitude of FRN is largely modulated by neural activity in gain trials. Monetary gain feedback has been proposed to elicit a distinct positive deflection, and reward positivity has been proposed to reflect dopaminergic signals in response to positive outcomes (Foti et al., 2014; Proudfit, 2015). Reframing FRN as a response to monetary gain (i.e., a neurobiological index of hedonic capacity) makes it well-suited for studying the motivational hierarchy in the motivational system. Reward positivity has been used to reliably measure reward sensitivity (Foti et al., 2014; Liu et al., 2014). Recent studies also detected an effect only in the win condition and not in the loss condition (Baker et al., 2016; Kessel et al., 2016). For example, a previous study found that participants were more sensitive to the win condition than to the loss condition (Yu and Zhang, 2014). Pathological gamblers manifest insensitivity to losses but hypersensitivity to wins (Hewig et al., 2010). In another study, a group of depressed individuals presented blunted responses to gain feedback compared with the control group, whereas no significant group difference emerged for loss feedback (Liu et al., 2014). Based on these data, we predicted that the influence of the motivational hierarchy on FRN would be significant in the win domain (reward positivity) and not in the loss domain.

Feedback-related negativity can be used as a valid electro-physiological marker for exploring levels of motivational significance. It is thought to be an earlier semiautomatic outcome evaluation process (Leng and Zhou, 2010; Zhou et al., 2010). This property of FRN makes it less susceptible to interference from social desirability than questionnaire methods. The gambling task is the most commonly used task to assess levels of motivation (Masaki et al., 2006). It has been used to compare motivational differences between the personal self and close others (Braams et al., 2014; Varnum et al., 2014; Zhu et al., 2015b). In the present study, our hypothesis was that the amplitude of reward positivity should reflect the hierarchical structure. Specifically, for Chinese college students, if close others have the same motivational hierarchy as the personal self, then reward positivity would not be able to differentiate between the personal self and close others. Conversely, if close others and the personal self have different motivational hierarchies, then reward positivity should be able to differentiate between these two groups.

## Materials and Methods

### Participants

Twenty-one college students (21.4 ± 0.8 years of age; range, 20–24 years; 10 females) participated in the study. The experiment was conducted in accordance with the Declaration of Helsinki and was approved by the Ethics Committee of the Department of Psychology, Henan University, China. Informed consent was obtained prior to the study. All of the participants had a normal or corrected-to-normal vision, and none had a history of neurological disease or brain injury. All of the participants were right-handed.

### Procedure

**FIGURE 1:**
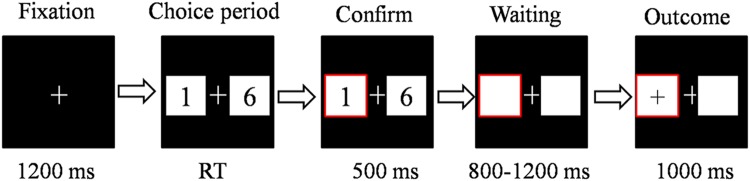
**The sequence of events within a single trial in the monetary gambling task.** In each trial, the beneficiary cue lasted for 3000 ms. The fixation point lasted for 1200 ms. The participant was then presented with a choice of two alternatives, and the participant responded using the left or right index finger. The alternatives remained until the participant made his/her choice. Afterward, his/her choice was highlighted for 500 ms. After a subsequent interval of 800–1200 ms, the participant received feedback, lasting 1000 ms, which indicated whether he/she gained or lost in that trial. RT, response time.

The participants underwent a simple gambling task (**Figure 1**). The gambling task was the same as in our previous study, with the exception that the numbers were changed (Zhu et al., 2015a) The stimulus display and behavioral data acquisition were performed using E-Prime 1.1 software (Psychology Software Tools). During the task, the participants sat comfortably in an electrically shielded room approximately 80 cm from a computer screen. Each trial began with 3000 ms presentation of the person for whom the participant was playing (i.e., “for yourself,” “for your mother,” “for your father,” “for your friend” and “for a stranger”). The participant was told that the strangers were selected from our subject pool. Two white rectangles (2.5°× 2.5° of visual angle) were then presented that contained two Arabic numerals (1 and 6, 2 and 7, 3 and 8, and 4 and 9) to indicate two alternative options on the left and right sides of a fixation point on the computer screen. The positions of the two numbers were counterbalanced across trials. The participants were asked to make a selection by pressing the “F” or “J” key on the keyboard with the left or right index finger, respectively. The alternatives remained on the screen until the participant chose one of the rectangles, which was then highlighted by a thick red outline for 500 ms. After a subsequent interval of 800–1200 ms, the participants received feedback, that lasted 1000 ms, and indicated whether he/she gained (when the valence of the outcome was “+”) or lost (when the valence of the outcome was “-”) in that particular trial (**Figure 1**). The formal task consisted of eight blocks of 80 trials each. Unbeknownst to the participants, the outcomes were provided according to a predetermined pseudorandom sequence, and each participant received exactly 64 of each kind of outcome for each beneficiary. Each participant was paid 15 CNY (~USD$2.3) for their participation in the study. In the gambling task, each beneficiary had 15 CNY in his/her account. Based on the points that were gained for each beneficiary, the final gain or loss was added to the separate account (every additional 500 points gained increased the payment by 5 CNY). Finally, the money was put on the close others’ or strangers’ cell phone. The total payment for each participant was approximately 75.6 CNY (range, 60–100 CNY; *SD* = 8.4 CNY).

Before the experiment, each participant was instructed about the rules and meaning of the symbols in the task. The participants were also encouraged to respond in such a way to maximize the total amount for each person. The participants were told that the beneficiary would receive more money at the end of the study if the participants earned more points. After the participant finished the task, he/she was told that the task had no optimal strategy.

### Electrophysiological Recording and Measures

Electroencephalographic (EEG) activity was recorded from 63 scalp sites using tin electrodes that were mounted in an elastic cap (Brain Products, Gilching, Germany) with an online reference to the FCz and off-line re-referenced to the average reference. Electrode FCz was re-instated (Zendel and Alain, 2014). The horizontal electrooculogram (HEOG) was recorded from an electrode that was placed at the outer canthi of the right eye. The vertical electrooculogram (VEOG) was recorded from an electrode that was placed above the left eye. All of the inter-electrode impedances were maintained at <10 kΩ. The EEG and EOG signals were amplified with a bandpass filter from 0.05 to 100 Hz and continuously sampled at 500 Hz/channel.

Off-line analysis of the EEG was performed using Brain Vision Analyzer software (Brain Products, Gilching, Germany). The first step in data preprocessing was the correction of ocular artifacts using Independent Component Analysis of the continuous data using Brain Vision Analyzer 2.0 software (Brain Products, Gilching, Germany). The ocular artifact-free EEG data were low-pass-filtered below 30 Hz (12 dB/oct) and high-pass-filtered above 0.1 Hz (12 dB/oct). Separate EEG epochs of 1000 ms (200 ms baseline) were extracted oﬄine for the stimuli. All of the trials in which EEG voltages exceeded a threshold of ±75 μV during the recording epoch were excluded from the analysis (~seven trials per condition were excluded).

The FRN amplitude was measured for each participant as the average amplitude within the 220–320 ms window (Zhu et al., 2015a). The electrode at which the FRN was detected was near the frontal midline (Fz, FCz, and Cz; Zhou et al., 2010). The FRN amplitudes were also entered into a 2 (feedback valence: win and loss) × 3 (electrode: Fz, FCz, and Cz) × 5 (beneficiary: personal self, mother, father, friend, and stranger) repeated-measures analysis of variance (ANOVA).

## Results

### Behavioral Results

For the gambling task, we defined the choices of 1, 2, 3, and 4 as the risk-avoidant choices in our experiment, predicting that the participants would make this choice to avoid the possibility of a large loss. However, by making these choices, they also lost the opportunity to receive the larger reward. In contrast, choosing larger numbers (6, 7, 8, and 9) was defined as the risky choice (high-risk, high-return). The one-way repeated-measures ANOVA revealed no main effect of motivational condition (self, mother, father, friend, and stranger) on the frequency of choosing the risky options [*F*(4,80) = 2.42, *p* = 0.11]. The repeated-measures ANOVA revealed no main effect of the beneficiary (self, mother, father, friend, and stranger) or size (small number vs. large number) on response time and no beneficiary × size interaction (all *ps* > 0.10).

### ERP Results

**FIGURE 2:**
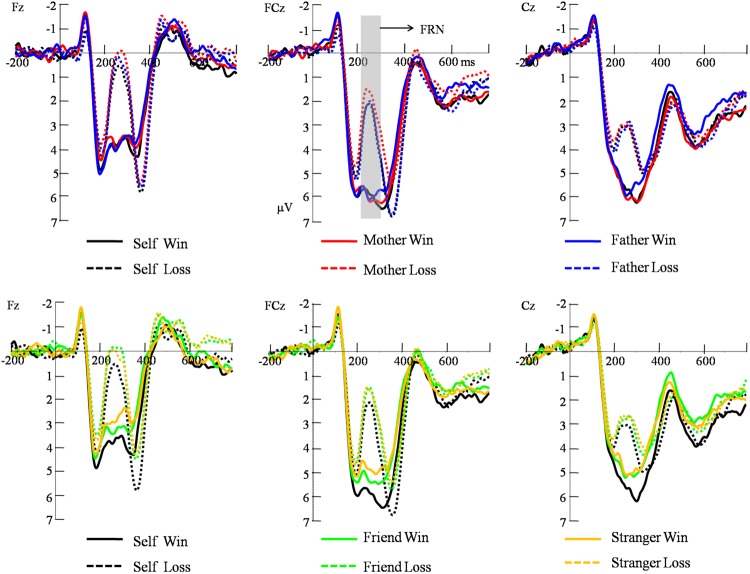
**Grand average FRN (feedback-related negativity) waveforms waves collapsed over reward magnitudes at three midline electrodes (Fz, FCz, and Cz) post-onset of the feedback stimuli.** The gray shaded areas indicate the FRN analysis window (220–320 ms) for average amplitudes.

For FRN (**Figure 2**), the main effect of electrode was significant [*F*(2,40) = 18.99, *p* < 0.001, η^2^ = 0.487], with a largest response at Cz site (*M* = 4.40 μV, *SE* = 0.45). The main effect of beneficiary was significant [*F*(4,80) = 4.85, *p* = 0.013, η^2^ = 0.195], with a largest response when gamble for self (*M* = 4.12 μV, *SE* = 0.46). The main effect of feedback valence was significant [*F*(1,20) = 117.27, *p* < 0.001, η^2^ = 0.85], such that losses evoked more negative response after (*M* = 2.75 μV, *SE* = 0.30) than after gains (*M* = 4.89 μV, *SE* = 0.44). The interaction between feedback valence and electrode was significant, [*F*(2,40) = 5.15, *p* = 0.018, η^2^ = 0.25]. The interaction between feedback valence and beneficiary was also significant, [*F*(4,80) = 2.93, *p* = 0.028, η^2^= 0.18]. The significant interaction was mainly due to the very small difference between Cz (5.42 μV) and FCz (5.58 μV; *p* = 0.528) in win condition and the difference between Cz (3.38 μV) and FCz (2.96 μV) was marginally significant in loss condition (*p* = 0.078). To further analyze the interaction between beneficiary and outcome valence, pair-wise analyses (Least Significant Difference test) revealed that there was no significant difference among the self and close others in the loss condition. In the win condition, the pair-wise analysis revealed the self (*M* = 5.27, *SE* = 0.52) evoked a reward positivity that was comparable to mother (*M* = 5.26, *SE* = 0.54) and father (*M* = 5.07, *SE* = 0.47; *p* > 0.1). The self, mother, and father evoked a larger reward positivity than friends (*M* = 4.62, *SE* = 0.40) and strangers (*M* = 4.24, *SE* = 0.37; *p*s < 0.05). Friends also evoked a larger reward positivity than strangers (*p* = 0.03) (see **Table 1**). The three-way interaction was not significant (*p* > 0.05).

**Table 1:** Mean reward positivity (standard error of the mean) during the gambling task.

	Win	Loss
Self	5.27 (0.52)	2.96 (0.42)
Mother	5.26 (0.54)	2.83 (0.30)
Father	5.07 (0.47)	2.82 (0.32)
Friend	4.62 (0.82)	2.61 (0.25)
Stranger	4.24 (0.75)	2.53 (0.32)

## Discussion

The present study investigated ERP responses to rewards in a social context, in which the personal self, close others, and strangers were the beneficiaries. Our main findings were threefold. First, the present results replicated the well-established ERP pattern in which losses evoked a more negative response than gains in a gambling task. Second, the reward positivity amplitudes that were evoked by winning were modulated by the beneficiary. Reward positivity did not differentiate between the personal self and parents. However, both the personal self and parents evoked larger reward positivity amplitudes than friends and strangers. Third, friends evoked larger reward positivity amplitudes than strangers.

In the present study, no differential reward positivity amplitude was found between parents and the personal self. For the mother, the present results were consistent with our previous study (Zhu et al., 2015b), which used the gambling paradigm and ERPs and found that the self and mother shared the same motivational hierarchy in the Chinese brain. The present study found that the father possesses the same motivational hierarchy as the personal self and mother. Previous fMRI studies found that the mPFC is less activated for father judgments than for self and mother judgments. Thus, mPFC activation in a trait judgment task was not a marker of different motivational hierarchies.

In the present study, the motivational hierarchy of friends was lower than the personal self, mothers, and fathers, but friends were still more important than strangers, indicating that friends are also deeply ingrained in the self motivational system. Generally, in Chinese culture, the union with family members is thought to be unconditional and unbreakable, whereas connections with friends can be fleeting (the notion of *Yuan*; Yang and Ho, 1988) and depend on reciprocal exchanges (the notion of *Renqin*; Yan, 1996). This result was consistent with a previous study of Chinese culture, in which Chinese individuals were found to value self-family connectedness more than self-friend connectedness (Li, 2002).

The present findings contrast with Kitayama and Park (2014), who used ERN as a neurological marker of motivation and found that it differentiated between the self and friends in Western culture but not in East Asian culture. Two methodological differences that may account for this discrepancy. First, reward positivity and ERN reflect different neural activity. The present study used reward positivity, which reflects the dopaminergic signal response to positive outcomes (Baker and Holroyd, 2011), whereas ERN is thought to index negative reward prediction errors that are based on a computation of an incorrect response as being worse than a correct response. Another reason is the that error response in the speeded conflict task (flanker task) is mainly due to ability, whereas winning or lossing in a gambling task mainly relies on luck. Therefore, it is likely that the motivation to do well is higher in the flanker task than in the gambling task. The motivation to do well in a task involves the anterior cingulate cortex (Bengtsson et al., 2009). The anterior cingulate cortex (ACC) is well known to be intimately involved in error detection (Van Veen and Carter, 2002). Enhanced ACC activity makes the ERN unable to differentiate between earning for the self and earning for a friend. The participants in the present study were presumed to feel safe while performing the gambling task (Hitokoto et al., 2016), which contributed to the ability to differentiate between gambling for the self and gambling for a friend.

Wang et al. (2012) suggested that globalization of the economy and education has allowed University students in China to be exposed to different cultural values and beliefs. The present results indicate that parents occupy the same motivational hierarchy as the personal self. The present results indicate that the family bond was still dominant in our participants who may be influenced by Western individualistic cultural values. However, a recent study found that overseas Chinese students failed to manifest overlapping representations between the self and mother (Chen et al., 2013). An interesting line of investigation would be to explore the motivational hierarchy in these overseas students to further clarify the ways in which cultural experiences influence the motivational hierarchy.

The present study has some limitations. First, we focused on the consummatory process in reward processing. An interesting line of investigation would explore anticipatory processing of the beneficiary cue when the participants know the beneficiary in the gambling task. Second, including a Western sample as a control group in the present study would have been informative. Cultural differences in FRN may be evident in this paradigm, corresponding to the notion that the personal self and parents have the same motivational hierarchy in Chinese culture but not in Western culture. Third, the present study employed a small sample size, future research with a larger sample (and hence greater statistical power) could involve validating the present results.

## Conclusion

The reward positivity response to gains in the gambling task provided evidence that the personal self and parents share a common hierarchy in the self-motivational system in the brains of Chinese individuals. Friends also occupied an important position in the self-motivation system but were less important than the self and parents.

## Author Contributions

XZ designed experiment and carried out experiment; LW, SY, and RG analyzed experimental results. HW and YL assisted with writing the manuscript. XZ wrote the manuscript.

## Conflict of Interest Statement

The authors declare that the research was conducted in the absence of any commercial or financial relationships that could be construed as a potential conflict of interest.
